# Uncovering the collateral impacts of COVID-19 on maternal mental health

**DOI:** 10.1186/s12978-022-01427-5

**Published:** 2022-05-11

**Authors:** Akaninyene Otu, Sanni Yaya

**Affiliations:** 1 Department of Microbiology, Leeds Teaching Hospitals NHS Trust, Leeds, UK; 2 School of International Development and Global Studies, University of Ottawa, 120 University Private, Ottawa, ON K1N 6N5 Canada; 3 The George Institute for Global Health, Imperial College London, London, UK

The COVID-19 pandemic has had a devastating impact on societal structures, health services and economies with over 40% of the world population infected at least once by Nov 14, 2021 [1]. Many communities have had to grapple with multiple stressors such as lockdowns, social distancing, debility, bereavement, and unemployment [2, 3]. Socioeconomic disparities resulting from job losses and great uncertainty about the future have been major issues experienced by many during this pandemic. These stressors have detrimentally impacted mental health with the World Health Organization (WHO) reporting a 25% increase in anxiety and depression in the first year of the COVID-19 pandemic [4]. Recent Global Burden of Disease data shows that COVID-19 has more severely affected the mental health of women more than men [5]. Evidence also points to a significant decline in maternal mental health during the COVID-19 pandemic [2, 5].

Perinatal mental health refers to mental health conditions affecting people during pregnancy and in the first year after giving birth. This includes a wide spectrum of conditions ranging from mild depression and anxiety to psychosis and recurrence of mental health issues during pregnancy [6, 7]. Research has shown that psychiatric admission is 22 times more likely following birth than in the pre-pregnancy period [8]. Conditions such as depression and anxiety are projected to affect one in seven women during the perinatal period, and carry an increased risk of preterm delivery, reduced mother-infant bonding, and impaired cognitive development of the infant [9]. Additionally, almost 20% of women diagnosed with postpartum depression have considered hurting themselves [10].

Even before COVID-19 emerged, perinatal mental disorders were the commonest complication of child‐bearing resulting in significant maternal and infant morbidity and mortality [11–13]. Perinatal mental health conditions are estimated to affect 10–20% of pregnancies although these differ by classification and disease severity [14]. Recent studies conducted in Italy [15], Canada [16], China [17] and Turkey [18] have reported a doubling in rates of depression and anxiety in pregnant women when compared to studies prior to the COVID-19 pandemic.

## Root causes and magnitude of the problem

As the true scale of the mental health burden of COVID-19 continues to emerge, it is now apparent that the restrictive measures such as quarantine, physical distancing, closure of schools and essential social services, have resulted in unprecedented stress on vulnerable groups within communities [19]. This is coupled with constraints on people’s ability to work and seek support from loved ones along with the ensuing financial worries [20]. Loneliness, fear of infection, grief after bereavement, health worker burnout are also stressors that have been linked to anxiety and depression following the emergence of COVID-19. Self-harm, harmful alcohol and drug use, insomnia, and suicidal behavior have reportedly risen on a global scale since the onset of the COVID-19 pandemic [21]. With families spending more time at home together during quarantine, an increase in cases of domestic violence was recorded among women who had no escape from their abusers [22–24].

The COVID-19 pandemic has exacerbated many of the recognized risk factors for maternal mental health disorders such as poverty, extreme stress, exposure to violence (domestic, sexual and gender-based) and low social support. This tenuous situation has been further worsened by the severe disruptions to mental health services and informal support, leading to huge gaps in care for vulnerable women who desperately require this. There are significant concerns that women with pre-existing mental health conditions could have been impacted negatively by these disruptions in service. Although virtual contact massively increased with mixed potential consequences there is a need to carefully evaluate the impact and outcomes of adopting virtual consultation. All this is further complicated by the data that shows that many women are disinclined to take antidepressants even when prescribed [25, 26].

Stigma is another major barrier which often prevents people from accessing care for maternal mental health disorders. Research has shown that women with mental health disorders present late for antenatal care due to the fear of stigma and judgmental attitudes of healthcare professionals [27].

Reproductive services have also been impacted negatively by COVID-19. Prior to the start of the COVID-19 pandemic, availability, access, and quality of interventions to address infertility was a challenge in most countries with women, poor, unmarried, uneducated and unemployed being disproportionately affected [28]. A systematic review of fifteen studies involving 5851 patients seeking fertility care unanimously concluded that the COVID-19 pandemic had a negative impact on fertility care [29]. Some of the risk factors included female sex, single marital state, prior diagnoses of anxiety or depression, and length of time trying to conceive.

A key barrier to the implementation of mental health services has been the perennial shortage of mental health workers, a problem which has persisted during the COVID-19 pandemic. In low and middle income countries (LMICs) approximately 85% of people with mental, neurological and substance-use (MNS) have been unable to access care [30] with the mental health work shortage estimated at estimated at 1.18 million mental health workers [31].

## Closing the huge gaps

The task of future-proofing perinatal mental health services against future public health crises is one that will clearly require cross-government approaches involving government departments, healthcare organizations, communities, and the voluntary sector. A good starting point would be to address the historic underinvestment in mental health services that has been seen globally. The commitment of governments worldwide needs to be secured to increase spending on mental health from the average of just over 2% of the health budget that was recorded in 2020 [21]. This increased spending can facilitate the training and recruitment of mental health workers to improve the situation in many LMICSs which have fewer than 1 mental health worker per 100,000 people. Specific legislations and frameworks are required, at national and local levels, to facilitate the prioritization of mental health especially during public health crises. An example of such legislation is the updated Comprehensive Mental Health Action Plan 2013–2030 which was adopted by member countries at the World Health Assembly in 2021 to improve preparedness for mental health and psychosocial support in public health emergencies.

The WHO has been at the forefront of the interagency mental and psychological response to COVID-19 which has involved partners such as other United Nations agencies, international nongovernmental organizations and the Red Cross and Red Crescent Societies. The WHO has promoted the prioritization and integration of mental health and psychosocial support into the global response to COVID-19. More work is required to maximize the output from such interagency initiatives.

The participation of voluntary and community organisations in providing maternal mental health support is vital. Such groups will be able to efficiently support women with maternal health problems while promoting health literacy and stigma reduction. The Maternal Mental Health Alliance (MMHA) in the United Kingdom is an example of a successful coalition of over 100 UK organisations, including professional bodies, that has functioned well during the COVID-19 pandemic to provide women with consistent, accessible and quality care for their mental health during pregnancy and after birth [32].

Specific measures are required to support the mental health of pregnant health workers who are more prone to suffering burnout which is a major trigger for suicidal thinking. Redeployment of such vulnerable staff away from patient-facing roles and the provision of resources to promote remote working can be beneficial. Access to specialist counsellors and support workers should be provided to such health workers. Further analysis of the efficacy of virtual consultations for maternal mental health is required. These virtual care services will need to be tailored and the adoption of hybrid models may help to increase access and address the issue of limited mental health staff at community level.

It is important to systematically tackle social discrimination, challenge negative perceptions and encourage open dialogue on mental health within communities. Social media and other digital tools could be leveraged to achieve this. A midwife-moderated social media based support service called Facemums was successfully used during the early stages of UK lockdown to support pregnant mothers on the Facebook social media platform [33]. Such platforms could be utilized to encourage women and indeed everyone with mental health disorders to seek help for these conditions [33].

The COVID-19 pandemic has laid bare deep-seated and perennial social vulnerabilities in society that are intricately linked to social determinants of health such as class, ethnicity, gender and education level. Practical measures that promote equity, social justice, and a shift in the balance of power and resources to people living in poverty are desirable and could improve the abysmal state of maternal mental health globally.

In conclusion, the significant decline in maternal mental health that has been recorded since the start of the COVID-19 pandemic needs to be urgently addressed. Multisectoral approaches that increase funding for mental health, alleviate social vulnerabilities and facilitate greater participation of community organizations holds promise for dealing with this formidable threat.

## References

[1] COVID-19 Cumulative Infection Collaborators, Estimating global, regional, and national daily and cumulative infections with SARS-CoV-2 through Nov 14, 2021: a statistical analysis. The Lancet. 2022. 10.1016/S0140-6736(22)00484-6. 10.1016/S0140-6736(22)00484-6 PMC8993157 35405084

[2] Jia R, Ayling K, Chalder T (2020). Mental health in the UK during the COVID-19 pandemic: cross-sectional analyses from a community cohort study. BMJ Open.

[3] Otu A, Charles CH, Yaya S (2020). Mental health and psychosocial well-being during the COVID-19 pandemic: the invisible elephant in the room. Int J Ment Health Syst.

[4] World Health Organization 2022. COVID-19 pandemic triggers 25% increase in prevalence of anxiety and depression worldwide. Available from: https://www.who.int/news/item/02-03-2022-covid-19-pandemic-triggers-25-increase-in-prevalence-of-anxiety-and-depression-worldwide. PMC9998057 35414629

[5] COVID-19 Mental Disorders Collaborators. Global prevalence and burden of depressive and anxiety disorders in 204 countries and territories in 2020 due to the COVID-19 pandemic. The Lancet 2021; 398 (10312): P1700–1712. 10.1016/S0140-6736(21)02143-7 PMC8500697 34634250

[6] Bailey L, Gaskin K (2021). Spotlight on maternal mental health: a prepandemic and postpandemic priority. Evid Based Nurs.

[7] O'Hara MW, Wisner KL (2014). Perinatal mental illness: definition, description and aetiology. Best Pract Res Clin Obstet Gynaecol.

[8] Kendell RE, Chalmers JC, Platz C (1987). Epidemiology of puerperal psychoses. Br J Psychiatry.

[9] Davenport MH, Meyer S, Meah VL, Strynadka MC, Khurana R (2020). Moms Are Not OK: COVID-19 and Maternal Mental Health. Front Global Women’s Health.

[10] MBRRACE-UK. MBRRACE-UK update: key messages from the UK and Ireland confidential enquiries into maternal death and morbidity 2018. Obstetr Gynaecol. 2019; 21:69–71. 10.1111/tog.12548.

[11] Maternal mental health alliance. The issue, perinatal mental illness. Available: https://maternalmentalhealthalliance.org/about/the-issue/ [Accessed 26 April 2022].

[12] Jones I, Chandra PS, Dazzan P (2014). Bipolar disorder, affective psychosis, and schizophrenia in pregnancy and the post-partum period. Lancet.

[13] Howard LM, Molyneaux E, Dennis CL (2014). Non-psychotic mental disorders in the perinatal period. Lancet.

[14] Stein A, Pearson RM, Goodman SH (2014). Effects of perinatal mental disorders on the fetus and child. Lancet.

[15] Saccone G, Florio A, Aiello F, Venturella R, Chiara De Angelis M, Locci M (2020). Psychological impact of coronavirus disease 2019 in pregnant women. Am J Obstet Gynecol.

[16] Lebel C, MacKinnon A, Bagshawe M, Tomfohr-Madsen L, Giesbrecht G (2020). Elevated depression and anxiety symptoms among pregnant individuals during the COVID-19 pandemic. J Affect Disord.

[17] Wu Y, Zhang C, Liu H, Duan C, Li C, Fan J (2020). Perinatal depressive and anxiety symptoms of pregnant women along with COVID-19 outbreak in China. Am J Obstet Gynecol.

[18] Durankuş F, Aksu E (2020). Effects of the COVID-19 pandemic on anxiety and depressive symptoms in pregnant women: a preliminary study. J Matern Neonatal Med..

[19] Singh S, Roy D, Sinha K, Parveen S, Sharma G, Joshi G (2020). Impact of COVID-19 and lockdown on mental health of children and adolescents: a narrative review with recommendations. Psychiatry Res.

[20] Yaya S, Otu A, Labonté R (2020). Globalisation in the time of COVID-19: repositioning Africa to meet the immediate and remote challenges. Global Health.

[21] World Health Organization. (2020c). Mental health and COVID-19. http://www.euro.who.int/en/health-topics/health-emergencies/coronavirus-covid-19/novel-coronavirus-2019-ncov-technical-guidance/coronavirus-disease-covid-19-outbreak-technical-guidance-europe/mental-health-and-covid-19.

[22] Abramson A. How COVID-19 may increase domestic violence and child abuse. American Psychological Association, April 8, 2020. https://www.apa.org/topics/covid-19/domestic-violence-child-abuse.

[23] Chandra J. Covid-19 lockdown | Rise in domestic violence, police apathy: NCW. The Hindu. 2020. https://www.thehindu.com/news/national/covid-19-lockdown-spike-in-domestic-violence-says-ncw/article31238659.ece.

[24] Graham-Harrison E, Giuffrida A, Smith H, Ford L. Lockdowns around the world bring rise in domestic violence. The Guardian. 2020. https://www.theguardian.com/society/2020/mar/28/lockdowns-world-rise-domestic-violence.

[25] Dennis CL, Chung-Lee L (2006). Postpartum depression help-seeking barriers and maternal treatment preferences: a qualitative systematic review. Birth.

[26] Chabrol HTF, Armitage J (2004). Acceptability of psychotherapy and antidepressants for postnatal depression among newly delivered mothers. J Reprod Infant Psychol.

[27] Moore D, Ayers S, Drey N (2017). The city MISS: development of a scale to measure stigma of perinatal mental illness. J Reprod Infant Psychol.

[28] World Health Organization. Infertility. Available from: https://www.who.int/news-room/fact-sheets/detail/infertility.

[29] Kirubarajan A, Patel P, Tsang J, Prethipan T, Sreeram P, Sierra S (2021). The psychological impact of the COVID-19 pandemic on fertility care: a qualitative systematic review. Hum Fertil.

[30] Endale T, Qureshi O, Ryan GK (2020). Barriers and drivers to capacity-building in global mental health projects. Int J Ment Health Syst.

[31] Kakuma R, Minas H, Ginneken N, Paz M, Desiiraju K, Morris J, Saxena S, Scheffler R (2011). Human resources for mental health care: current situation and strategies for action. Lancet.

[32] Maternal Mental Health Alliance. COVID-19 information and support from Maternal Mental Health Alliance members. Available from: https://maternalmentalhealthalliance.org/news/mmha-members-offer-reassurance-amid-coronavirus-outbreak/.

[33] Chatwin J, Butler D, Jones J (2021). Experiences of pregnant mothers using a social media based antenatal support service during the COVID-19 lockdown in the UK: findings from a user survey. BMJ Open.

